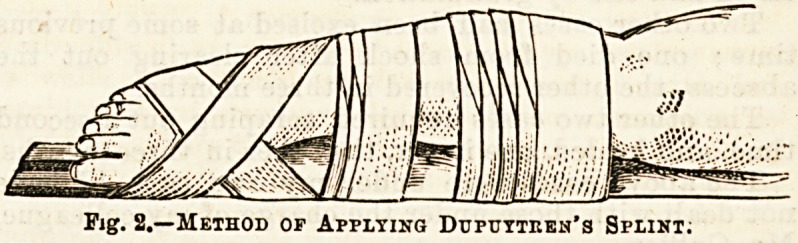# The Treatment of Pott's Fracture

**Published:** 1893-01-28

**Authors:** 


### THE BIRMINGHAM HOSPITALS.

### The Treatment of Pott's Fracture.

Fractures of the tibia and fibula in the vicinity of the ankle joint, besides forming a large proportion of the fractures met with in hospital work, occur so frequently in private practice as to render the consideration of their treatment a matter of interest to the general practitioner.

Inasmuch as the practical import of such injuries is, to a great extent, quite independent of mere anatomical details, we here follow the prevailing laxity in the usage of the term, "Pott's Fracture," and include under that one heading all the fractures involving the malleolar ends of the tibia and fibula.

In this region four varieties of fracture are to be met with.

I. That originally described by Percival Pott, and from which he himself suffered, namely, fracture of the fibula, about three inches from the malleolus externus, with separation of the tip of the malleolus internus, and accompanied by eversion, with more or less backward displacement of the foot.

II. Fracture of the fibula in its lower third, with rupture of the internal lateral ligament.

III. Fracture involving the lower third of both the tibia and fibula.

IV. Fracture of the internal malleolus and rupture of the external lateral ligament.

For the purposes of treatment, however, these varieties arrange themselves under two headings.

A. No marked deformity; or, if present, is easily reduced.

B. Characterised by displacement, which offers some resistance to reduction.

In the diagnosis of these injuries, the possibility of error can only arise in those cases where there is no manifest deformity apart from the swelling which helps to mask the signs. Careful examination shoul d however, elicit some, if not all, the usual diagnostic features; thus, in the absence of distinct crepitus, the loss of elasticity in the continuity of the bone, together with a localised painful area to which pain is referred when pressure is exerted at a distance, should sufficiently indicate the nature of the injury.

In the treatment of these fractures, we may first consider those cases where deformity does not exist, or has been easily reduced. In these cases trouble may arise from the great swelling which occurs, especially if the patient is not seen until some time after the accident. It is, however, quite unnecessary to apply local absorbents; for no agent is so potent in dispersing such swelling as perfect immobilisation of the affected part. The surgeon may therefore proceed at once to apply whatever apparatus he may have selected.

At the Birmingham hospitals three methods alone are now in vogue: (*a*) Starch apparatus; (*b*) mill-board lateral splints; (*c*) Croft's modification of the Bavarian splint.

To apply the starch case, the limb is first well protected by layers of cotton wool rolled evenly from the toes to a little above the knee, the foot being held by an assistant firmly at a right angle with the axis of the leg. Next to the wool are placed three strips of mill-board softened in warm water, and about two and a half inches wide, and long enough to reach from the sole of the foot to just above the knee; one is placed at the back of the leg and one on either side. Over these are then rolled muslin bandages soaked in boiled starch, and in applying them it is important to leave the toes quite exposed, to avoid reversing the bandage, to avoid pulling the bandage, only exerting just sufficient traction to form even turns; and, lastly, to take extra turns around the ankle so as to give greater support to that part where movement is most liable to take place. The leg is then slung up by dry bandages to some form of cradle, and hot bottles placed on either side the limb to hasten the process of drying. At the end of about twelve hours the case may be cut up in front in order to avoid any possibility of constriction of the limb, and an ordinary roller bandage applied around it. The chief objection to the starch apparatus is the slowness with which it dries.

FIG. 1.

The second method, and one which is very largely adopted here, is the application of lateral splints cut out of mill-board. The limb having been previously well wrapped round with cotton wool, and then these splints are fixed by an ordinary roller bandage, and the limb steadied in bed by sandbags placed on either side. As soon as all swelling has subsided, an outer casing of plaster of Paris bandages is added, and the patient may be allowed to get about, provided the limb is not used to support the weight of the body until after a period of seven weeks, when firm union having been obtained the splint may be discarded.

Lately Croft's modified Bavarian splint has been largely used in the treatment of these fractures; and the cheapness and ease with which the necessary materials may be obtained, causes it to be a method of great value to the general practitioner.

To apply this apparatus four pieces of house flannel are roughly cut out to the shape of the limb, the width of each being half an inch less than half the circumference of the limb at the corresponding level. Two of these are required to form the outer splint, and two for the inner splint. The limb is first protected by a soft flannel bandage, then two of the pieces are laid out on a table (as in Fig. 1), and the other two pieces of flannel are dippep into plaster of Paris, mixed with water to the consistence of thin cream, and then each is placed in contact with its

FIG. 2.—METHOD OF APPLYING DUPUYTREN'S SPLINT.

fellow that has not been dipped; by this means the one surface of the splints so formed is devoid of plaster, and this surface is placed next to the bandaged limb, and an ordinary roller bandage is rapidly carried round the splints so as to mould them to the shape of the limb. The splints become dry in about ten minutes, and the limb immobilised.

In the treatment of those fractures associated with marked displacement which resists attempts at reduction, by far the most satisfactory method is to place the patient under an anæsthetic, and having brought the displaced fragments into good apposition, at once fix the limb securely by one of the forms of plaster cases. Various splints have been suggested to correct such displacements; thus, Dupuytren's splint applied to the inner side of the limb was formerly the plan adopted for reducing the eversion of the foot. (Fig. 2.)

For the backward dislocation, flexion of the knee with the limb laid on its outer side, so as to relax the traction of the calf muscles, has been one method adopted, and in severe cases division of the tendo Achillis. The Anlerio horse-shoe splint and Roughton's splint have also had their advocates, but these are gradually and deservedly falling into disuse, and the treatment of fractures at the present day is marked by the uniformity and simplicity in the methods adopted.

Should stiffness of the ankle joint remain after the splints have been removed, massage of the foot will restore the mobility of the joint.

## Figures and Tables

**Fig. 1. f1:**
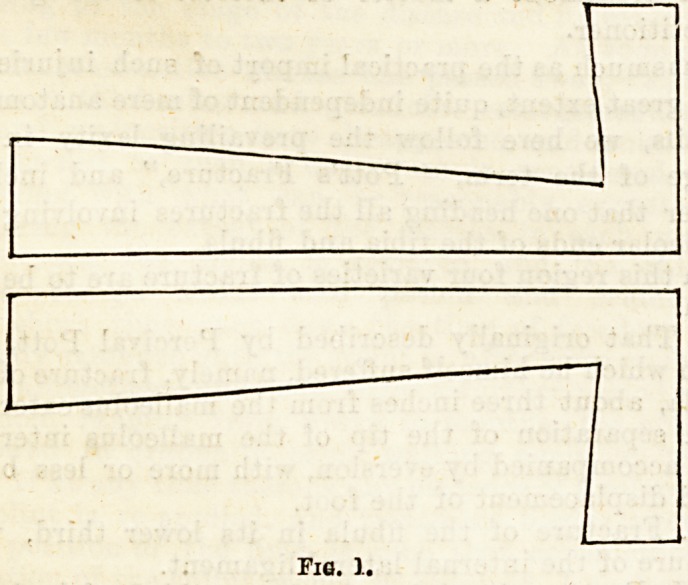


**Fig. 2. f2:**